# Development of a New Portable Genetic Analyzer for Point‐of‐Care Molecular Genetics and Pharmacogenomics Analysis

**DOI:** 10.1155/humu/9614566

**Published:** 2026-07-12

**Authors:** Ioanna Poulida, Kariofyllis Karamperis, Ioanna Konstantina Routsi, Ioannis Sarris, George Mantzouranis, Vassilis Kostopoulos, Christina Mitropoulou, George P. Patrinos

**Affiliations:** ^1^ School of Health Sciences, Department of Pharmacy, Laboratory of Pharmacogenomics and Individualized Therapy, University of Patras, Patras, Greece, upatras.gr; ^2^ The Golden Helix Foundation, London, UK; ^3^ Polytechnic School, Department of Mechanical Engineering and Aeronautics, University of Patras, Patras, Greece, upatras.gr; ^4^ Laboratory of Innovative Therapeutics and Personalized Medicine, Hellenic Pasteur Institute, Athens, Greece, pasteur.gr; ^5^ Department of Genetics and Genomics, College of Medicine and Health Sciences, United Arab Emirates University, Al-Ain, UAE, uaeu.ac.ae; ^6^ Clinical Bioinformatics Unit, Department of Pathology, Faculty of Medicine and Health Sciences, Erasmus University Medical Center, Rotterdam, the Netherlands, erasmusmc.nl

**Keywords:** 3D printing, *β*-thalassemia, CYP2C19, HBB, molecular diagnostics, personalized medicine, pharmacogenomics, point-of-care (PoC)

## Abstract

Traditional medicine is now moving from the “one‐size‐fits‐all” model toward personalized medicine, where diagnostic and therapeutic decisions are guided by the patient’s unique genetic profile. Recent advances in genomics and pharmacogenomics have facilitated the identification of genetic variants linked to disease susceptibility and progression, as well as variability in drug response. However, translating these findings into clinical practice remains challenging, primarily due to the high cost and sophisticated genetic analysis infrastructure, which is only available in centralized genetic laboratories. A newly developed Portable Genetic Analyzer (PortaGen) was designed for point‐of‐care molecular genetics and pharmacogenomics analysis and evaluated in this study. PortaGen integrates 3D‐printed parts and laptop‐based centralized software control, along with digital recording and storage of results to support decentralized genetic testing. The prototype portable device was validated in comparison with an established portable polymerase chain reaction (PCR) workstation that complies with current operational standards and a reference laboratory‐based method. Genotyping analysis was performed using ARMS‐PCR (Amplification Refractory Mutation System Polymerase Chain Reaction) to detect and analyze *CYP2C19* genetic variants (*CYP2C19* ∗ *2; rs4244285*, and *CYP2C19* ∗ *17*; *rs12248560*), relevant to pharmacogenomics, as well as the *HBB: c.93-21(G>A)* genetic variant, the most common variant leading to *β*‐thalassemia. Concordance in genotyping calls between the new device, the established portable workstation, and the reference method was assessed using percentage agreement and Cohen’s kappa coefficient, demonstrating consistently high concordance with statistically significant results (p < 0.05). These findings demonstrate that the new portable genotyping analyzer has improved throughput, visualization, and workflow efficiency into a suitcase‐sized, portable point‐of‐care molecular genetic analysis device, which holds promise to advance personalized medicine interventions in a scalable and affordable fashion.

## 1. Introduction

Medicine is shifting towards personalization, integrating clinical indices with genetic information, environmental exposures, and patient lifestyle factors [1]. Advances in genomics, multiomics, pharmacogenomics (PGx), bioinformatics and molecular diagnostics (MDx), such as next‐generation sequencing technologies, have enabled the detection of genomic variants. It is well established that the interpretation of genetic information contributes to the prediction of disease risk and progression, while enabling rational therapeutic strategies [1–3]. If implemented at scale, a personalized medicine approach promises real‐world clinical benefits for both patients and the healthcare system as a whole. This approach enhances disease prevention and early detection while positively influencing treatment adherence, improving therapeutic efficacy and reduced drug‐related toxicity. Beyond individual patient benefits, this paradigm advances population health, productivity and delivers potential cost benefits for healthcare systems and society [1, 4].

Nowadays, the majority of MDx technologies remains centralized and mostly relies on trained personnel and specialized laboratory facilities. Consequently, in remote areas, this requires sample collection and transport to central laboratories or hospitals, leading to extended turnaround times and higher associated costs. The required instrumentation is often expensive and demands regular maintenance, which makes routine laboratory testing that is standard in developed countries costly and inaccessible to patients and clinicians in remote and resource‐limited settings. These barriers underscore the urgent need for portable, affordable, and user‐friendly MDx tools to enable broader implementation of personalized medicine [5, 6].

More recently, point‐of‐care (PoC) MDx devices have emerged as a promising solution to overcome the limitations described above. These systems can deliver reliable results from MDx analysis near the site of patient care by combining portability, rapid, robust testing, and simplified workflows [7]. In line with the World Health Organization’s (WHO) ASSURED criteria, effective PoC devices should be Affordable, Sensitive, Specific, User‐friendly, Rapid and Robust, Equipment‐free, and Deliverable to end users [8]. For example, PoC nucleic acid amplification systems have demonstrated clear clinical value in diverse applications, particularly in infectious diseases through rapid detection of pathogens such as SARS‐CoV‐2 and HIV in decentralized and resource‐limited settings [9, 10]. In PGx, multiple *CYP2C19* genotyping tests are already available and adopted in clinical trials, reflecting the clinical importance of actionable variants that influence clopidogrel activation and patients’ drug response [11, 12]. Recent research efforts are extending PoC diagnostics beyond infectious diseases and PGx to MDx of genetic disorders like *β*‐thalassemia and cystic fibrosis, among others, aiming for faster screening and diagnosis outside traditional laboratories [13–15].

A portable MDx device that brings together thermal cycling, microcentrifugation, gel electrophoresis, and visualization of electrophoresis results in a single compact workstation is BentoLab [16, 17]. BentoLab has been used in rapid onsite workflows for detecting SARS‐CoV‐2 RNA from environmental surface swabs [18], as well as in PGx testing [19]. In addition, this portable device can be combined with nanopore sequencing technology to move beyond simple detection toward full genomic analysis right on site [20, 21].

Despite the growing availability of portable MDx devices, important challenges remain regarding analytical throughput, workflow integration, and adaptability to different genetic testing applications. Many existing systems are designed for specific assays or offer limited sample processing capacity, which may restrict their use in broader PGx and MDx settings. Therefore, there remains a need for PoC diagnostic devices that combine multiple laboratory functions within a single workstation while supporting increased analytical throughput and flexibility for diverse targeted genetic analyses [16, 22].

Building on advances in PoC MDx, a newly developed portable analyzer, PortaGen, was designed to support decentralized genetic testing through the integration of thermal cycling, microcentrifugation, gel electrophoresis, and result visualization within a single portable platform. In addition to consolidating these laboratory functions, the system was developed to support higher sample throughput and parallel processing workflows, enabling its application in both PGx and MDx analyses.

## 2. Materials and Methods

### 2.1. Development of the New Portable Genetic Analyzer

**Figure 1:**
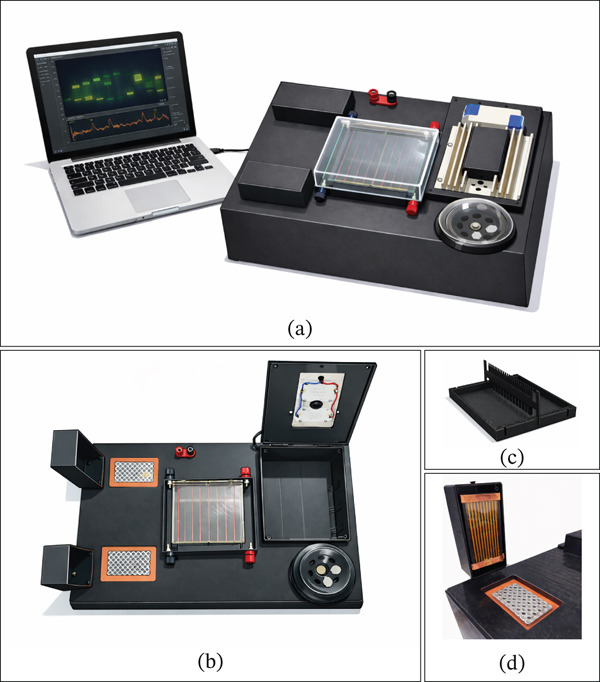
Illustration of the PortaGen prototype portable genotyping device and key components (21.5 cm H × 35.5 cm W × 45.5 cm L). (a) PortaGen in an operational setup connected to a laptop for centralized control and digital recording of results, shown with the module lids closed. (b) Top view of the PortaGen device shown with the lids open, indicating the layout of the integrated modules: the 3D‐printed gel electrophoresis chamber is positioned centrally, the UV LED‐based gel visualization module is located on the right, the two 40‐well thermocyclers are positioned on the left, and the 3D‐printed microcentrifuge unit is located on the lower right. (c) 3D‐printed gel casting comb/tray kit used for agarose gel preparation. (d) One of the two independent 40‐well thermal blocks equipped with a heated lid for PCR reactions.

The newly developed PortaGen device integrates the main instrumentation needed for routine genetic analysis, including thermocycling, microcentrifugation, gel electrophoresis, and UV visualization into a single portable analyzer operated through a centralized laptop‐based software interface (Figure 1). PortaGen incorporates two independent 40‐well PCR blocks and an electrophoresis apparatus with a UV LED‐based gel visualization module. A high‐resolution camera within the UV LED‐based module enables gel visualization and automated result storage. Key parts, like the electrophoresis chamber and the microcentrifuge, were fabricated using 3D printing technology, to support affordable device production.

To the best of our knowledge, PortaGen represents one of the first portable platforms to introduce these features, constituting a notable technical advancement in PoC genotyping. PortaGen is designed to increase practical throughput and minimize turnaround time. It allows for either concurrent processing of up to 80 samples or parallel PCR protocols using two independent 40‐well blocks, while also supporting electrophoresis of up to 52 samples per gel run. Additionally, the centralized laptop‐based control system, which has an easy‐to‐use interface, simplifies workflow and reduces handling errors. Real‐time thermal performance curves (set‐point vs. measured temperature) enable verification of PCR cycling accuracy, supporting reproducible amplification and reliable genotyping results. For electrophoresis result interpretation, PortaGen offers multiple visualization modes (standard color, contrast‐enhanced for common DNA stains, intensity‐based rendering, and noise‐reduced enhancement). These visualization options help with clear gel documentation, improved band detectability, and fewer ambiguous genotype calls, while maintaining traceable records that can be reviewed by multiple operators (Figure S1).

### 2.2. Study Sample

In total, 101 individuals from two independent cohorts were included in this study, comprising a PGx cohort (*n* = 51) and a MDx cohort (*n* = 60). Participants in the PGx cohort were enrolled in the PREPARE (PREemptive Pharmacogenomic testing for preventing Adverse drug REactions) study, a prospective, open‐label, randomized controlled clinical trial conducted at the Laboratory of Pharmacogenomics and Individualized Therapy, Department of Pharmacy, University of Patras, Greece. The PREPARE study was implemented within the framework of the European Commission–funded Ubiquitous Pharmacogenomics (U‐PGx) project [23, 24]. The MDx cohort included *β*‐thalassemia patients from Western Greece who were recruited for the assessment of the molecular spectrum of *β*‐thalassemia in the region [25].

The PGx cohort was genotyped for the *CYP2C19* ∗ *2* (*CYP2C19* c.681G>A; *rs4244285*) and *CYP2C19* ∗ *17* (*CYP2C19* −806C>T; *rs12248560*) genetic variants, corresponding to a loss‐of‐function and a gain‐of‐function allele, respectively, which are clinically relevant for clopidogrel bioactivation and therapeutic response [26]. The MDx cohort referred for diagnostic testing and was tested for the *HBB*: c.93‐21(G>A) [*IVSI-110* (G>A), *rs35004220*] variant, a common *β*‐thalassemia associated allele in the Mediterranean region [27]. Written informed consent was obtained from all participants before enrolment, and the study was conducted in accordance with the protocol approved by the Institutional Ethics Committee of the University of Patras.

### 2.3. Genetic Analysis

Genomic DNA was extracted from peripheral whole blood leukocytes using the NucleoSpin Blood kit (Macherey‐Nagel), based on silica membrane spin‐column technology, following the manufacturer’s instructions. DNA was eluted in a final volume of 50 *μ*L, yielding concentrations typically ranging from 40 to 100 ng/*μ*L. DNA concentration and purity were measured using a microvolume UV–Vis spectrophotometer (Quawell, Q6000). Samples with A260/280 ratios within the acceptable range (~1.8–2.1) and adequate DNA concentration (> 40 ng/*μ*L) were retained for downstream analyses.

Genetic analysis was carried out using a standardized ARMS‐PCR workflow across the BentoLab and PortaGen. PCR amplification was performed with DF Taq polymerase and reagents (EnzyQuest) and allele‐specific primer sets were designed to selectively amplify the *CYP2C19* ∗ *2*, *CYP2C19* ∗ *17* and *IVSI-110* (G>A) genetic variants (detailed protocols available upon request). The ARMS‐PCR protocols allow precise distinction of the wild‐type, heterozygous, and homozygous alternate genotypes. Fragment patterns corresponding to each genotype were visualized by agarose gel electrophoresis and used to assign genotype calls for each sample on both portable devices. ARMS‐PCR experiments were performed on BentoLab and PortaGen in parallel using identical protocols, primer sets, and reagents to ensure comparability between devices.

In accordance with the study design, genotyping performance was evaluated by concordance analyses, including comparisons of portable device genotype calls with the reference genotypes for the genetic variants with available reference data, as well as direct pairwise comparisons between the two portable devices for all evaluated genetic variants. For the PGx cohort (*CYP2C19* ∗ *2* and *CYP2C19* ∗ *17*), ARMS‐PCR genotype calls from BentoLab and PortaGen were each compared with the corresponding reference genotypes (BentoLab vs. Gold‐Standard Method; PortaGen vs. Gold‐Standard Method), and PortaGen calls were additionally compared with BentoLab under identical ARMS‐PCR conditions (PortaGen vs. BentoLab). For these PGx variants the reference method was Restriction Fragment Length Polymorphism PCR (RFLP‐PCR) in which *CYP2C19* regions harboring the ∗ *2* and ∗ *17* variants were amplified by PCR, followed by variant‐specific restriction enzyme digestion and visualized by agarose gel electrophoresis. For the MDx cohort (*IVSI-110*(G>A) variant), performance was assessed through agreement between PortaGen and BentoLab using the same standardized ARMS‐PCR workflow under matched experimental conditions.

### 2.4. Validation and Comparative Assessment of Portable Genotyping Platforms

In this step, t*ο* facilitate comparative validation, results derived from the reference method and the two portable genotyping devices (BentoLab and PortaGen) were numerically encoded for each genetic variant as follows: 1 for homozygous wild‐type allele, 2 for heterozygous genotype, and 3 for homozygous alternate allele. Following genotype encoding, categorical agreement analysis between two methods each time was performed using overall percentage agreement and unweighted Cohen’s kappa (*κ*). Cohen’s kappa statistics were implemented in R using the *irr* package (Version 4.5.2), with genotype classes (wild‐type, heterozygous, and homozygous alternate variant) treated as nominal and equally distinct categories. The resulting coefficients were interpreted according to standard thresholds (0.00–0.20 = *none*, 0.21–0.39 = *minimal*, 0.40–0.59 = *weak*, 0.60–0.79 = *moderate*, 0.80–0.90 = *strong*, and > 0.90 = *almost perfect agreement*) [28].

To support visual interpretation of concordance patterns, pairwise agreement heatmaps were generated to display agreement and disagreement in genotype classifications across comparisons using the *ggplot2* package in R (version 4.0.1). Additionally, Venn diagrams were created as a summary of overlap in genotype calls across the two portable devices and the reference method using the *VennDiagram* package in R (Version 1.8.2).

Additional classification performance metrics were calculated in R (version 4.5.2). Confusion matrices were generated for each pairwise comparison, and overall accuracy was calculated as the proportion of correctly classified samples relative to the total number of samples analyzed. False‐positive rates (FPR) and false‐negative rates (FNR) were derived from the confusion matrices using a one‐vs‐rest approach for each genotype category (wild‐type, heterozygous, and homozygous alternate genotype), whereby each genotype class was considered the positive class and the remaining genotype classes were grouped as the negative class.

### 2.5. Variant Annotation, Genotype Frequency and Quality Control Analysis

To ensure accurate mapping, rsID‐specific queries were performed, and the most relevant annotation record was selected based on exact rsID matching. Missing annotations were retained as such, reflecting the absence of corresponding predictive information rather than technical failure.

Importantly, PGx clinical guidelines for *CYP2C19* genetic variants were not queried at the individual variant level, as such guidelines are defined at the gene–drug–phenotype level rather than per rsID. Instead, variants were interpreted in the context of established star allele definitions and their contribution to metabolizer phenotypes with known clinical relevance.

As a complementary analysis, genotype distributions derived from reference method and portable devices were evaluated separately for each genetic variant for consistency with Hardy–Weinberg equilibrium (HWE) using the chi‐square (*χ* ^2^) test, performed with the *HardyWeinberg* R package (Version 1.7.9). Within this analytical framework, observed genotype frequencies were compared with reference population data using Fisher’s exact test. Reference population‐based genotype frequencies for the *CYP2C19* variants were obtained from the Ensembl database (GRCh38.p14 human genome assembly) [29]. For the *IVSI-110*(G>A) variant, population reference frequencies were retrieved from the NCBI dbSNP Allele Frequency Aggregator (ALFA) dataset (Release 4; release Version 20250407153717) [30]. These reference data were used to assess whether the study cohorts were consistent with expected population distributions and did not exhibit major deviations. All statistical analyses were conducted in R (Version 4.5.2; R Foundation for Statistical Computing, Vienna), using a combination of packages (*HardyWeinberg*, *httr (1.4.7)*, *jsonlite (2.0.0)*, *purrr(1.2.0)*, and *dplyr(1.1.4)*).

## 3. Results

### 3.1. Preliminary Operational Performance of the PortaGen Genotyping Device

Prior to comparative assessment, referring to genotyping efficiency and accuracy, our new portable genetic analyzer successfully met all predefined functional and performance tests, demonstrating stable and consistent operation under the applied experimental conditions. Thermal cycling was reliable across the full temperature range required for PCR (up to 40 cycles) to support efficient target amplification. Moreover, performance specifications remained within acceptable limits for genotyping applications and were sufficient to support reliable genotype calling. Notably, the system was evaluated at full operational capacity, processing a total of 80 samples simultaneously, across two independent 40‐well PCR blocks, with successful amplification and clearly distinguishable genotype‐specific banding patterns using the integrated electrophoresis module and UV LED‐based gel visualization module of the device (Figure 1). Collectively, these results confirmed the suitability of the portable system for downstream comparative evaluation against established laboratory‐based genotyping platforms.

### 3.2. Analytical Performance of Reference Method and Portable Devices

Complete genotype calls were obtained for all PGx (*CYP2C19* ∗ *2* and *CYP2C19* ∗ *17)* and MDx (*IVSI-110*(G>A)) participants using the reference method and both portable devices (BentoLab and PortaGen). Overall, genotype distributions were broadly consistent with HWE. Fisher’s exact tests showed no significant differences between the observed genotype distributions and population reference frequencies for any variant (Table S1).

### 3.3. High Concordance of BentoLab Genotyping Results With the Gold‐Standard Method

To assess BentoLab as a benchmark portable device, ARMS‐PCR genotyping calls for *CYP2C19* generated on BentoLab were compared with genotypes obtained using the reference method. For *CYP2C19* ∗ *2,* BentoLab showed high overall agreement with the reference method, with a percentage of 96.1%. The Cohen’s kappa coefficient was 0.889 (*p* = 9.33 × 10^−14^), indicative of strong concordance beyond chance. For the *CYP2C19* ∗ *17* variant agreement was 92.2% and Cohen’s kappa was 0.853 (*p* = 3.70 × 10^−13^) reflecting again strong concordance (Figure S2).

Across both PGx variants, discordances were mainly shifts between wild‐type and heterozygous calls, with no misclassification of homozygous alternate reference genotypes. Together, these results indicate that BentoLab generates genotype calls that are highly consistent with the reference method, supporting its suitability as a reliable comparator for evaluating PortaGen performance.

### 3.4. High Concordance of PortaGen Genotyping Results With the Gold‐Standard Method

ARMS‐PCR genotype calls generated on PortaGen showed high concordance with the reference method for the *CYP2C19* variants. For *CYP2C19* ∗ *2*, PortaGen achieved a percentage agreement of 94.1% with the gold‐standard method. Cohen’s *kappa* was 0.845 (*p* = 6.47 × 10^−13^) and is consistent with strong concordance according to standard interpretation thresholds. Similarly, for *CYP2C19* ∗ *17*, PortaGen achieved a percentage agreement of 90.2% and Cohen^’^s Kappa = 0.819 (*p* = 9.89 × 10^−13^) indicating strong concordance.

**Figure 2:**
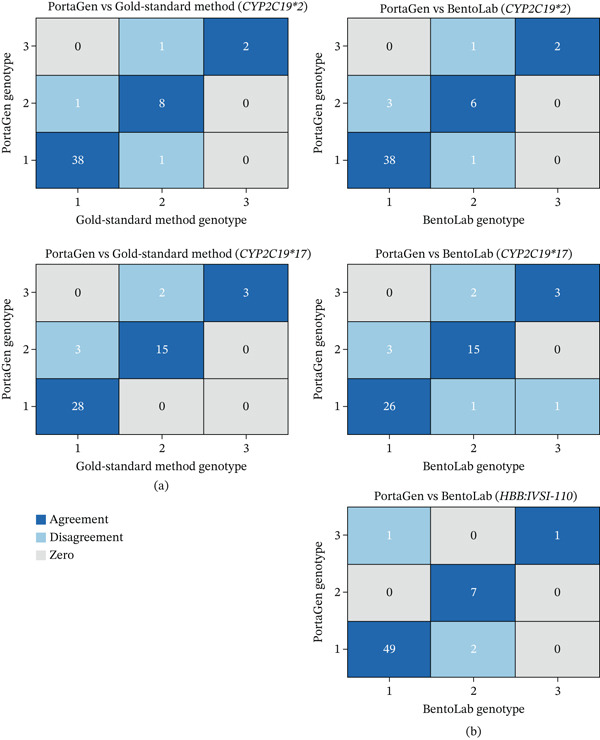
Agreement heatmaps comparing the *CYP2C19* ∗ *2*, *CYP2C19* ∗ *17*, and *IVSI-110 (G>A)* genotype calls between (a) the PortaGen and Gold‐Standard method, and (b) PortaGen and BentoLab portable devices. Cells display the number of samples classified into each genotype combination (1: homozygous to wild‐type allele, 2: heterozygous, 3: homozygous to alternate allele). Dark blue cells denote concordant genotype classifications (agreement), light blue cells denote discordant classifications (disagreement), and light grey cells indicate comparisons with zero observations.

Overall, PortaGen showed robust, non‐chance genotype classification relative to the reference method as reflected by strong percent agreement and Cohen’s Kappa values. Pairwise concordance between PortaGen and the gold‐standard method is summarized in the agreement heatmaps. The few discordant calls were mainly wild‐type to heterozygous genotype calls shifts, with only occasional heterozygous to homozygous alternate differences (Figure 2a).

### 3.5. Comparative Genotyping Agreement Between the PortaGen and the BentoLab Portable Workstations

The performance of the PortaGen was assessed by direct comparison with the validated portable PCR workstation, BentoLab. For the *CYP2C19* ∗ *2* variant, the PortaGen demonstrated 90.2% overall agreement with the BentoLab. The Cohen’s kappa coefficient was 0.724 (*p* = 4.39 × 10^−10^), corresponding to moderate concordance. For the *CYP2C19* ∗ *17*, agreement was 86.3% with Cohen’s kappa of 0.753 (*p* = 3.65 × 10^−11^) again corresponding to moderate concordance. Evaluation of the *IVSI-110*(G>A) variant was based on a pairwise comparison of genotype calls between the two portable platforms. For this genetic variant, PortaGen achieved 95.0% overall agreement with the BentoLab, demostrating strong concordance beyond chance (Cohen^’^s Kappa = 0.817, *p* = 3.97 × 10^−13^).

Across all variants, an agreement between PortaGen and BentoLab was consistently high, with discordant calls restricted to a small subset of samples. Most discrepancies reflected adjacent genotype‐class shifts, primarily between wild‐type and heterozygous calls, with only occasional heterozygous to homozygous alternate differences and rare wild‐type to homozygous alternate mismatches. These patterns are also summarized in the agreement heatmaps (Figure 2b).

### 3.6. Overall Quantitative and Qualitative Concordance of Genotyping Results Across All Systems

**Figure 3:**
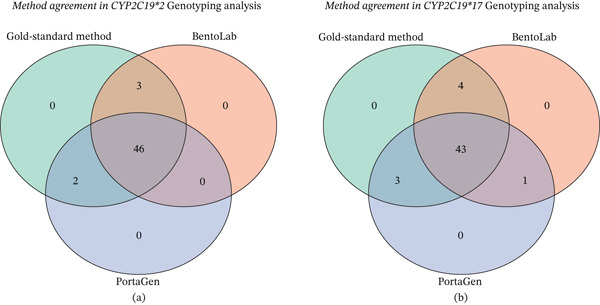
Venn diagrams show the overlap of genotype assignments for (a) *CYP2C19* ∗ *2* and (b) *CYP2C19* ∗ *17* among the gold‐standard method, BentoLab, and PortaGen in the PGx cohort (*n* = 51). Numbers denote the count of samples sharing identical genotype calls within each intersection. The central region represents concordant calls across gold‐standard method and portable devices, whereas the remaining regions represent calls shared by only two methods or unique to a single method, indicating discordant classifications.

To summarize, the two portable devices, PortaGen and BentoLab, produced highly concordant genotype calls across clinically relevant variants. BentoLab showed close alignment with the reference method, supporting its role as an established portable workstation. The prototype PortaGen demonstrated mainly strong agreement with both the reference method and the BentoLab. The discordances were limited to a small number of samples. The agreement heatmaps further reflect these concordance patterns, supporting PortaGen’s analytical performance for PoC genotyping (Figure 2). Indicatively, Venn diagrams were presented to illustrate overlap in *CYP2C19* genotype calls between the reference method, BentoLab and PortaGen. From the Venn diagrams, most samples were assigned the same *CYP2C19* genotype across the reference method and both portable devices, indicating strong overall agreement (46/51 patients for *CYP2C19* ∗ *2* and 43/51 patients for *CYP2C19* ∗ *17*) (Figure 3).

Additional classification performance analysis demonstrated overall accuracies of 94.1%, 90.2%, and 95.0% for *CYP2C19* ∗ *2*, *CYP2C19* ∗ *17*, and *IVSI-110*(G>A), respectively (Table 1). Across all evaluated assays, PortaGen correctly classified 151 of 162 samples, corresponding to an overall analytical accuracy of 93.2%. Genotype‐specific FPR and FNR were overall low (Table S2). Most discordant classifications involved adjacent genotype categories (wild‐type and heterozygous or heterozygous and homozygous alternate genotypes), while homozygous alternate genotypes exhibited consistently high classification performance across all evaluated assays.

**Table 1:** Analytical performance of PortaGen across the evaluated PGx and MDx assays. Overall analytical accuracy was assessed as the proportion of correctly classified samples relative to the total number of samples analyzed, while agreement with the comparator method was evaluated using unweighted Cohen’s kappa (*κ*).

Assay	Comparator	Samples (*n*)	Correct Calls	Accuracy (%)	Cohen’s kappa
*CYP2C19* ∗ *2*	Gold‐standard	51	48	94.1	0.845
*CYP2C19* ∗ *17*	Gold‐standard	51	46	90.2	0.819
*IVSI-110 G>A*	BentoLab	60	57	95.0	0.817
Overall	—	162	151	93.2	—

**Figure 4:**
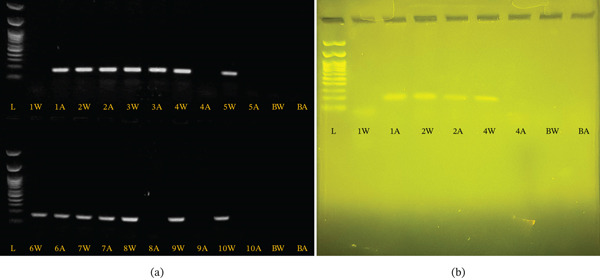
Representative ARMS‐PCR electrophoresis results obtained with (a) PortaGen and (b) BentoLab are shown, where L denotes the 100‐bp DNA ladder and, for each patient, W and A correspond to the wild‐type and alternate alleles. BW and BA indicate the no‐template (blank) controls for the W and A reactions. Based on band presence, Patient 1 shows amplification only in the A lane, consistent with a homozygous alternate genotype, Patients 2 and 3 show amplification in both W and A lanes, consistent with a heterozygous genotype and Patients 4 and 5 show amplification only in the W lane, consistent with a homozygous wild‐type genotype. Visualization results are superior using the PortaGen UV LED‐based module, as the green background shown in Panel (b) makes visualization of the PCR results more difficult.

To complement these quantitative agreement analyses, representative ARMS‐PCR electrophoresis images are presented to confirm that genotype‐specific banding patterns were visually consistent across PortaGen and BentoLab (Figure 4). In both portable devices, the expected fragment patterns for the *CYP2C19* ∗ *2*, *CYP2C19* ∗ *17*, *IVSI-110* (G>A) variants enabled clear visual distinction between homozygous to wild‐type allele, heterozygous genotype, and for homozygous to alternate allele. In addition, the electrophoresis setup supported higher per‐run sample visualization on PortaGen (up to 10 patients per run) compared with BentoLab (up to three patients per run). Differences in gel background between imaging setups affected contrast and could influence the ease of band visualization. Notably, visualization was clearer with the PortaGen UV LED‐based module, whereas the green background in BentoLab reduced contrast and made PCR bands more difficult to interpret.

## 4. Discussion

Portable diagnostic devices are expected to play an important role in expanding the use of Personalized Medicine in remote areas and resource‐limited settings. By combining multiple laboratory functions within a single workstation while supporting increased analytical throughput and flexibility for diverse targeted genetic analyses, these user‐friendly devices hold promise for cost‐effective and accurate PGx and MDx testing services.

The findings of this study indicate that portable genotyping devices like PortaGen can achieve high concordance with reference methods. This emphasizes their analytical reliability for PoC genetic testing. PoC genetic analysis devices play a vital role in advancing the goals of personalized medicine. These devices can provide clinically relevant genetic information at the PoC, potentially improving access to genetic testing in remote and resource‐limited settings. In fact, the clinical utility of these devices has already been explored for both PoC genome‐guided therapy (bedside *CYP2C19* PGx testing to guide antiplatelet therapy) and infectious disease diagnostics in decentralized areas [6, 31].

In line with this approach, proof‐of‐concept and implementation studies in cardiology demonstrate the feasibility of bedside *CYP2C19* genotyping analysis for guiding antiplatelet therapy shortly after percutaneous coronary intervention (PCI) [31, 32]. At the same time, evaluations of integrated cartridge‐based devices like Genomadix (formerly Spartan RX) show that accurate *CYP2C19* genotyping analysis is possible with limited hands‐on molecular processing. This supports the concept that automation of genetic analysis workflow can reduce training requirements and practical barriers for bedside PGx testing [11, 22, 33]. In line with these clinical applications, the observed analytical concordance, accuracy, and agreement metrics support the potential of PortaGen as a portable PoC tool for *CYP2C19* PGx testing.

Implementation of PoC genetic testing faces important challenges. Initially, many existing devices are built around single‐gene or single‐disease MDx, limiting scalability as personalized medicine shifts towards multi‐gene PGx panels and broader genetic screening strategies [23, 34]. In contrast, PortaGen is not restricted to a single genetic target. In this study, ARMS‐PCR protocols were standardized for two clinically relevant PGx variants in *CYP2C19* and for an MDx variant (*IVSI-110*(G>A)) in the *HBB* gene. These findings suggest that additional assay standardization on PortaGen may be feasible, which could broaden its applicability across PGx testing, infectious and genetic disease MDx. Although, because of the proof‐of‐principle nature of our study, we restricted our analysis to small variant panels for MDx of monogenic diseases and PGx analysis, this device can also accommodate analysis of larger variant panels and even structural variants. In particular, *CYP2D6* expanded PGx variant panels that also include structural variants often used in psychiatry, can be efficiently analyzed in the PortaGen device.

Beyond the ability of PoC testing to support multiple genetic targets, additional practical barriers limit wider adoption. In some cases, analytical performance is inferior to central laboratory methods, particularly when sample preparation is simplified or devices are highly miniaturized [11, 35]. Moreover, limited digital result capture and poor integration with electronic health records can reduce the long‐term clinical value of genotype results, especially for PGx data that should remain accessible for future genome‐guided therapy decisions [36, 37]. Several PoC devices also depend on complex workflows and expert interpretation of results, which can affect reliability outside specialized laboratories, especially when they rely on proprietary, manufacturer‐specific reagents and closed consumables [38]. In this context, PortaGen was designed to address several of these challenges while remaining grounded in familiar molecular biology procedures (PCR, centrifugation, and electrophoresis). It uses standard reagents and conventional protocols, maintaining reliable analytical performance. Digital control and imaging of PortaGen support workflow automation, improved visualization, and result archiving. This combination positions PortaGen between traditional benchtop approaches and fully cartridge‐based PoC genotyping devices.

In addition to their analytical and operational characteristics, portable MDx devices can also play an important role in raising genomics education and general public awareness. The BioSTEM initiative has shown that combining online modules with hands‐on experiments using a suitcase‐sized molecular biology workstation (BentoLab) can significantly improve students’ and educators’ understanding of genetics, PGx and personalized medicine, and can be deployed even in remote schools and outreach events. Such activities help demystify genetic testing to the public, highlight both the potential and limitations of genomics, and support the development of a potential workforce of healthcare personnel familiar with PGx and MDx techniques [39]. In a similar way, PortaGen could serve a dual purpose, as a practical tool for decentralized genetic testing in clinical or remote settings and as a training device for laboratory staff and students, supporting hands‐on education in PoC genetic analysis workflows. In addition, it can promote public understanding of personalized medicine and PGx.

Overall, this study contributes to the ongoing efforts to improve PoC genetic testing devices by demonstrating that a portable, streamlined approach can support clinically relevant genotyping analysis across more than one genetic variant. However, the present evaluation of PortaGen was limited to three genomic variants in two well‐defined but relatively restricted cohorts. Despite these limitations, the analysis remained robust, with strong concordance observed in the majority of comparisons. At the same time, the observed discordances in a small number of samples underline the need for broader validation in larger and more diverse populations, as well as careful attention to robustness under clinical and nonclinical conditions. Furthermore, although agreement, accuracy, FPR, and FNR were evaluated, additional performance characteristics such as intra‐run reproducibility, inter‐run reproducibility, hands‐on time, and time‐to‐result were not systematically assessed and therefore remain important objectives for future validation studies. Additionally, PortaGen is primarily suited for targeted applications (small number of genetic variant sets for PGx and MDx) rather than much broader multi‐variant panels. These results provide a solid foundation for further evaluation and optimization of PortaGen for routine and decentralized use.

In conclusion, the PortaGen device demonstrated high overall analytical performance, achieving strong concordance and high classification accuracy across the evaluated assays while supporting genotyping analysis in a portable and compact device. PortaGen can generate accurate and high‐quality genotyping results, demonstrating strong concordance with the gold‐standard method and an established portable PCR workstation. In addition, it offers high‐throughput operation, allowing multiple samples to be processed in parallel and enabling several workflow steps to be performed simultaneously. This can streamline the overall analysis workflow and reduce hands‐on time. Importantly, the PortaGen design relies on well‐established and broadly applicable principles of MDx while using accessible and affordable consumables. This approach strengthens PortaGen’s potential for adoption across diverse laboratories, remote areas, and resource‐limited settings. With further validation across additional genetic targets and larger cohorts, PortaGen has strong potential to evolve into a practical PoC genotyping tool for routine and decentralized applications, while also serving as an educational device.

## Funding

This study was supported by The Golden Helix Foundation; University of Patras, 10.13039/100009043. The publication of this article in OA mode was financially supported by HEAL‐Link.

## Conflicts of Interest

The authors declare no conflicts of interest.

## Supporting information


**Supporting Information** Additional supporting information can be found online in the Supporting Information section.

## Data Availability

Data are available from the corresponding author upon reasonable request.
