# Supplementary material for: Development of a New Portable Genetic Analyzer for Point‐of‐Care Molecular Genetics and Pharmacogenomics Analysis
Source: Hum Mutat. 2026 Jul 12;2026:9614566. doi: 10.1155/humu/9614566 (PMC13357681; doi:10.1155/humu/9614566)
Supplement: Supplementary file 1 — Supporting Information Additional supporting information can be found online in the Supporting Information section. [file HUMU-2026-9614566-s001.docx]

**Supplementary Information**

**Development of a new portable genetic analyzer for point-of-care molecular genetics and pharmacogenomics analysis**

**Ioanna Poulida ^1^, Kariofyllis Karamperis ^1,3^, Ioanna Konstantina Routsi ^1^, Ioannis Sarris ^2^, George Mantzouranis ^2^, Vasileios Kostopoulos ^2^, Christina Mitropoulou ^3^, George P. Patrinos ^1,4,5,6,^***

^1^ University of Patras, School of Health Sciences, Department of Pharmacy, Laboratory of Pharmacogenomics and Individualized Therapy, Patras, Greece

^2^ University of Patras, Polytechnic School, Department of Mechanical Engineering and Aeronautics, Patras, Greece

^3^ The Golden Helix Foundation, London, UK

^4^ Hellenic Pasteur Institute, Laboratory of Innovative Therapeutics and Personalized Medicine, Athens, Greece

^5^ Department of Genetics and Genomics, College of Medicine and Health Sciences, United Arab Emirates University, Al-Ain, United Arab Emirates

^6^ Clinical Bioinformatics Unit, Department of Pathology, Faculty of Medicine and Health Sciences, Erasmus University Medical Center, Rotterdam, the Netherlands

**Supplementary Figure 1. Representative ARMS-PCR gel image capture and software visualization outputs from PortaGen.** Agarose gel electrophoresis results are shown in multiple viewing modes generated by the PortaGen imaging software: (a) standard color capture, (b) contrast enhanced for common DNA stains, (c) contrast-enhanced/noise-reduced view to improve band visibility, and (d) intensity-based (3D) rendering of band signal.

**
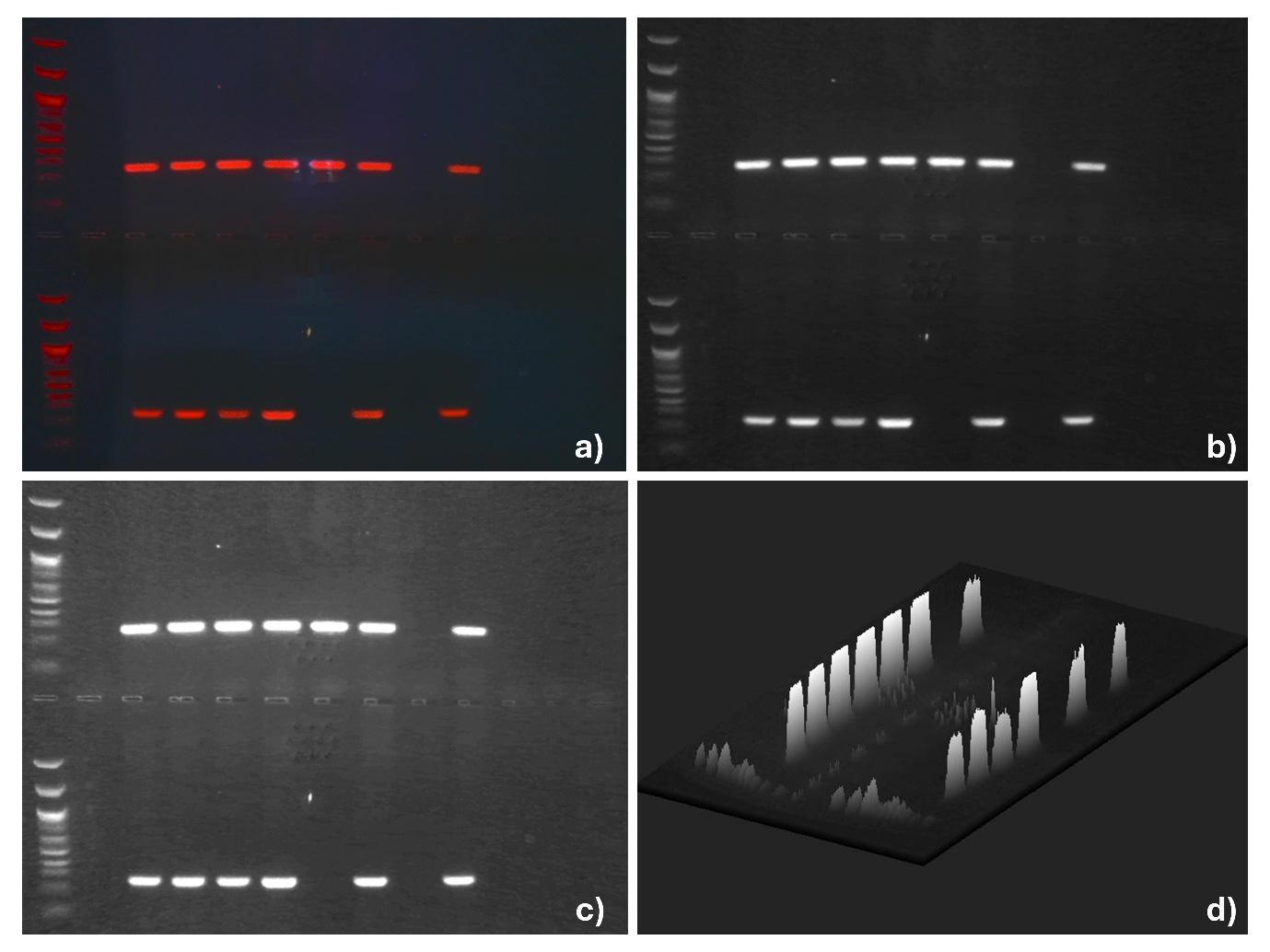
**

**Supplementary Figure 2. Agreement heatmaps comparing the *CYP2C19*2*, *CYP2C19*17* genotype calls between the BentoLab and Gold-Standard method.** Cells display the number of samples classified into each genotype combination (1: homozygous to wild-type allele, 2Q heterozygous, 3: homozygous to alternate allele). Dark blue cells denote concordant genotype classifications (agreement), light blue cells denote discordant classifications (disagreement), and light grey cells indicate comparisons with zero observations.

**
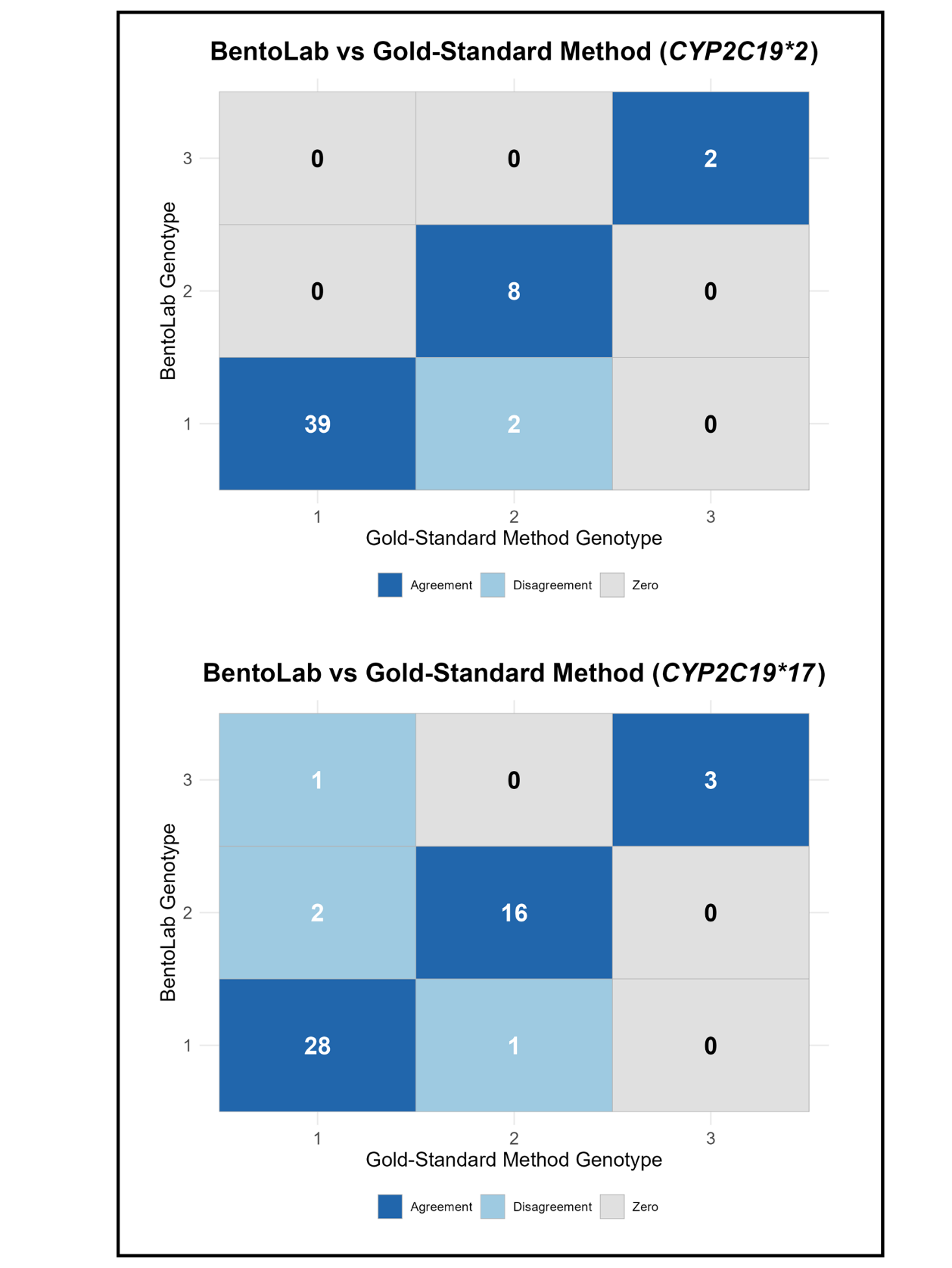
**

**Supplementary Table 1.** Observed allele frequencies, Hardy-Weinberg equilibrium (χ², p), and Fisher exact test results (p) for each evaluated variant across the reference method, BentoLab, and PortaGen.

| **SNP** | **Method** | **HWE**  **(χ^2^, (p))** | **Fisher test (p)** | **WT Allele (%)** | **Alternate Allele (%)** |
| --- | --- | --- | --- | --- | --- |
| ***CYP2C19*2*** | **Reference (RFLP-PCR)** | 1.51 (0.2191) | 0.7630 | 86.3 | 13.7 |
|  | **BentoLab (ARMS-PCR)** | 3.05 (0.0809) | 0.5687 | 88.2 | 11.8 |
|  | **PortaGen (ARMS-PCR)** | 4.49 (0.0342) | 0.4134 | 85.3 | 14.7 |
| ***CYP2C19*17*** | **Reference (RFLP-PCR)** | 0.11 (0.7443) | 0.5883 | 77.5 | 22.5 |
|  | **BentoLab (ARMS-PCR)** | 0.26 (0.6129) | 0.4339 | 74.5 | 25.5 |
|  | **PortaGen (ARMS-PCR)** | 0.66 (0.4160) | 0.2354 | 72.5 | 27.7 |
| ***IVSI-110* G/A** | **BentoLab (ARMS-PCR)** | 0.59 (0.4420) | 0.0748 | 90.8 | 9.2 |
|  | **PortaGen (ARMS-PCR)** | 5.38 (0.0204) | 0.1470 | 90.8 | 9.2 |

**Supplementary Table 2.** Genotype-specific false-positive rates (FPR) and false-negative rates (FNR) for PortaGen across the evaluated pharmacogenomic and molecular diagnostic assays. FPR and FNR were calculated from confusion matrices using a one-vs-rest approach for each genotype category, including homozygous wild-type (HOM WT), heterozygous (HET), and homozygous alternate (HOM ALT) genotypes. For each analysis, the genotype category under evaluation was considered the positive class, while the remaining genotype categories were grouped as the negative class.

| **Variant** | **Genotype** | **FPR (%)** | **FNR (%)** |
| --- | --- | --- | --- |
| ***CYP2C19*2*** | HOM WT | 8.3 | 2.6 |
|  | HET | 2.4 | 20.0 |
|  | HOM ALT | 2.0 | 0.0 |
| ***CYP2C19*17*** | HOM WT | 0.0 | 9.7 |
|  | HET | 8.8 | 11.8 |
|  | HOM ALT | 4.2 | 0.0 |
| ***IVSI-110 G/A*** | HOM WT | 20.0 | 2.0 |
|  | HET | 0.0 | 22.2 |
|  | HOM ALT | 1.7 | 0.0 |
